# Commentary: Derivation of Simian Tropic HIV-1 Infectious Clone Reveals Virus Adaptation to a New Host

**DOI:** 10.3389/fcimb.2020.00235

**Published:** 2020-05-15

**Authors:** Akio Adachi, Takaaki Koma, Naoya Doi, Masako Nomaguchi

**Affiliations:** ^1^Department of Microbiology, Kansai Medical University, Osaka, Japan; ^2^Department of Microbiology, Tokushima University Graduate School of Medical Science, Tokushima, Japan

**Keywords:** SIVmac, *vif* gene, macaque-tropic HIV-1, adaptive mutations, pig-tailed macaques, rhesus macaques

HIV-1 is strictly tropic for humans with respect to its replication and pathogenicity. This striking biological property of HIV-1 limits our animal model systems for various basic and clinical investigations (Hatziioannou et al., 2006; Kamada et al., 2006; Ambrose et al., 2007; Nomaguchi et al., 2008, 2011; Thippeshappa et al., 2011, 2012; Hatziioannou and Evans, 2012; Misra et al., 2013). Among animal models tested, the best one that amply and most correctly reflects the final outcome of the HIV-1-human interaction as well as the initial and intermediate processes would be the HIV-1-macaque infection system (Hatziioannou and Evans, 2012). Non-human primates frequently used for experimental virus infection are rhesus, cynomolgus, and pig-tailed macaques. Of these, the rhesus macaque is the best characterized and the most utilized host species in infection models for HIV-1/AIDS research (Hatziioannou and Evans, 2012). Three major cellular factors that restrict HIV-1 replication in macaque cells, designated restriction factors (Malim and Bieniasz, 2012), are well-documented to be TRIM5 (Stremlau et al., 2004), APOBEC3 (Sheehy et al., 2002), and tetherin/BST-2 (Neil et al., 2008; Van Damme et al., 2008) proteins. In human cells, HIV-1 Gag-capsid (CA), Vif, and Vpu circumvent the anti-viral activities imposed by the orthologs of macaque TRIM5, APOBEC3, and tetherin, respectively (Malim and Bieniasz, 2012). Of note, pig-tailed macaques do not express TRIM5 that restricts HIV-1 replication. In order to successfully establish the macaque experimental system for HIV-1/AIDS studies, researchers including the authors of the titled article have been working on this issue by modifying HIV-1 to be tropic for macaques through subtle alterations in its genome (alterations in *gag*-CA, *vif* , and/or *vpu*) (Hatziioannou et al., 2006, 2014; Kamada et al., 2006; Kuroishi et al., 2009; Thippeshappa et al., 2011; Nomaguchi et al., 2013b; Soll et al., 2013). Needless to mention, pathogenic molecular clones are essential for studying viral spread/distribution/transmission in individuals and/or populations, viral mutation/adaptation/evolution under variable environments, and finally the disease progression in a precise and perspective manner. Resultant macaque-tropic HIV-1 derivative clones were tested for their *in vivo* activity in pig-tailed (Igarashi et al., 2007; Hatziioannou et al., 2009; Thippeshappa et al., 2011), cynomolgus (Saito et al., 2011), and rhesus (Doi et al., 2018) macaques. So far, no viral clones that persist in infected macaques long enough to cause AIDS have been reported except for that in this titled work.

Authors of the titled article have succeeded in generating a molecular clone pathogenic for pig-tailed macaques. Their study to generate the clone was as follows. Viruses derived from a molecular clone carrying the SIVmac *vif* gene (designated stHIV-1) were inoculated into pig-tailed macaques, which lack anti-TRIM5 activity. Recovered viruses from the animals were then repeatedly transferred from CD8^+^ cell-depleted animal-to-animal until the pathogenic viruses appeared. They have obtained a viral swarm that can cause AIDS in transiently CD8^+^ cell-depleted pig-tailed macaques (Hatziioannou et al., 2014). Inoculation of viruses from a certain animal with AIDS into naïve animals did not cause AIDS, but did in CD8^+^ cell-depleted animals. From the virus pool derived from this animal, a molecular clone designated stHIV-A19 was generated and demonstrated to consistently cause AIDS in CD8^+^ cell-depleted pig-tailed macaques as detailed in the titled article. While CD8^+^ cell-depletion is still required, this is the first molecular clone that rapidly cause AIDS in macaques. Because stHIV-A19 has the authentic HIV-1 CA and pig-tailed macaques lack virus-restrictive TRIM5, it is possible by this system to study interaction of HIV-1 CA and cellular factors other than TRIM5 in the course of pathogenic infection *in vivo*.

During studies on construction and characterization of macaque-tropic HIV-1 derivative clones, numerous intriguing observations related to viral variation, mutation, adaptation, and evolution were noted (Nomaguchi et al., 2013a,c, 2014a, 2016, 2017; Thippeshappa et al., 2013; Yokoyama et al., 2016; Doi et al., 2019a,b; the titled article, 2019). These issues are relevant to fundamental viral properties, and provide research subjects critical for basic virology. We have performed extensive virus adaptation experiments using cynomolgus/rhesus macaque cell lines (Akari et al., 1999; Doi et al., 2011) and prototype macaque-tropic HIV-1 derivative clones (Kamada et al., 2006; Nomaguchi et al., 2008) to improve their poor replication potentials in macaque cells. The macaque cell lines used display the phenotype exquisitely similar to that of peripheral blood mononuclear cells (PBMCs) in regard of sensitivity to virus infection and of virus productivity upon infection. Results obtained have indicated that HIV-1 derivatives effectively adapt to grow better in new host cells by mutations, especially those in the regulatory region for *vif* gene (SA1D2prox) (Nomaguchi et al., 2013a, 2014a, 2016, 2017) and coding-region of *env* gene (Kamada et al., 2006; Nomaguchi et al., 2013a; Yokoyama et al., 2016; Doi et al., 2019b). Thippeshappa et al. has shown that a variant HIV-1 derivative, more resistant to the restriction by interferon α than the prototype, grows better in pig-tailed macaque CD4^+^ cells (Thippeshappa et al., 2013). Finally, the authors of the titled article have demonstrated that their pathogenic stHIV-1 (stHIV-A19) can adaptively mutate during its replication in pig-tailed macaques to acquire new CAs, which are partially resistant to a cellular anti-viral factor Mx2.

In conclusion, a new molecular clone of the HIV-1 derivative virus that can cause AIDS in transiently CD8^+^ cell-depleted pig-tailed macaques has been successfully generated as fully described in the titled paper. However, it still fails to overcome CD8^+^ T-cell response. Considering the high level of polymorphism in the infected pig-tailed macaques and also as a main target of CTL, Nef might deserve more attention, in addition to Gag, to generate pathogenic clones. As for the rhesus macaque models, we do not have such molecular clones as yet. While even our best clones (Nomaguchi et al., 2013b, 2014b) can grow fairly well in cultured cell lines and macaque PBMCs, they are unable to persist and cause the disease in rhesus macaques (Doi et al., 2018). Further studies are required to generate molecular clones that can cause AIDS in naïve pig-tailed and rhesus macaques.

## Author Contributions

AA conceived the idea and wrote the manuscript. TK, ND, and MN reviewed and modified the manuscript. All authors approved its submission.

## Conflict of Interest

The authors declare that the research was conducted in the absence of any commercial or financial relationships that could be construed as a potential conflict of interest.
